# Clinical characteristics and frequent risk factors in young adult patients with stroke at a tertiary care hospital in Honduras

**DOI:** 10.3389/fstro.2026.1887056

**Published:** 2026-07-23

**Authors:** Kivian Obed Matamoros Padilla, Roberto Daniel Padilla Valeriano, Eleonora Espinoza-Turcio, Henry Noel Castro-Ramos, Lysien Ivania Zambrano, Selvin Z. Reyes-Garcia

**Affiliations:** 1 Postgraduate Program of Neurology, Faculty of Medical Sciences, National Autonomous University of Honduras (UNAH), Tegucigalpa, Honduras; 2 Department of Internal Medicine, Faculty of Medical Sciences, National Autonomous University of Honduras (UNAH), Tegucigalpa, Honduras; 3 Faculty of Medical Sciences, National Autonomous University of Honduras (UNAH), Tegucigalpa, Honduras; 4 Group GRINVAR, Faculty of Medical Sciences (FCM), National Autonomous University of Honduras (UNAH), Tegucigalpa, Honduras; 5 Mental Health Research Group, Faculty of Medical Sciences (FCM), National Autonomous University of Honduras (UNAH), Tegucigalpa, Honduras; 6 Department of Morphological Sciences, Faculty of Medical Sciences (FCM), National Autonomous University of Honduras (UNAH), Tegucigalpa, Honduras; 7 Faculty of Medical Sciences (FCM), Institute for Research in Medical Sciences and Health Rights (ICIMEDES), National Autonomous University of Honduras (UNAH), Tegucigalpa, Honduras

**Keywords:** foramen ovale patent, Honduras, stroke, ultrasonography, Doppler, transcranial, young adult

## Abstract

**Background:**

Stroke has become the third leading cause of death, accounting for 10% of all deaths. The prevalence of intracranial hemorrhage, subarachnoid hemorrhage, and ischemic stroke has increased by 20.5%, 11.5%, and 7.4%, respectively, in the 20–54 age group, making it a worldwide problem for the young adult population.

**Aim:**

To describe the clinical characteristics, diagnostic findings, stroke subtypes, and frequent risk factors among young adult patients with stroke treated at a tertiary care hospital in Honduras.

**Methods:**

Cross-sectional, non-probability convenience sample study of 70 patients aged 18–55 years. An ethical opinion was obtained (079-2024). The information was supplemented with clinical examinations, laboratory tests, neuroimaging, carotid Doppler, and transcranial Doppler with bubble test. Descriptive statistics were obtained.

**Results:**

Young adults accounted for 30.0% of stroke cases evaluated. The mean age was 43.4 ± 9.1 years. The 61.4% were women, 55.7% had a history of hypertension, 31.4% had type 2 diabetes mellitus, and 24.3% had migraines. A total of 21.4% were smokers, and 15.7% of women used oral contraceptives. Ischemic stroke occurred in 71.4% of cases, with a cardioembolic etiology in 21.4%. A patent foramen ovale (PFO) was identified in 5 of 17 cases, corresponding to 29.4% of the tested subgroup and 7.1% of the total sample and mortality was 15.7%.

**Conclusions:**

A high proportion of strokes were observed in women, with a predominance of ischemic strokes of cardioembolic etiology. Modifiable factors associated with stroke were identified, which can be controlled, contributing to a decrease in morbidity and mortality from this pathology.

## Introduction

Given the scarcity of local data on young-onset stroke in Honduras and Central America, hospital-based studies may provide valuable preliminary evidence to characterize clinical profiles, identify modifiable risk factors, and inform future regional surveillance and prevention strategies.

Stroke is defined as the presence of acute signs and symptoms of focal brain injury that persist for 24 h or more, or that cause death within 24 h (updated in CM-11, 2022) (WHO, 2022).

In 2019, there were 12.2 million (95% *CI*: 11.0–13.6) incident cases of stroke, 101 million (93.2–111) prevalent cases, 143 million (133–153) disability-adjusted life years (DALYs) attributable to stroke, and 6.55 million (6.00–7.02) deaths from this cause (Feigin et al., 2021). In 2021, stroke was the third leading cause of death worldwide, accounting for 10% of all deaths (WHO, 2024).

The incidence of stroke by age in high-income countries shows divergent results, with less favorable trends in younger age groups compared to older ones (Scott et al., 2022). Figures have been reported indicating that up to 14% of all ischemic strokes occur in young adults (<55 years) (Schaapsmeerders et al., 2013).

A meta-analysis by Soto Venegas (2024) reported a sex-adjusted stroke prevalence in Europe of 9.2% (95% *CI*: 4.4–14.0); in men it was 9.1% (95% *CI*: 4.7–16.6) and in women it was 9.2% (95% *CI*: 4.1–14.4). The sex-adjusted stroke incidence was 191.9 per 100,000 person-years (95% *CI*: 156.4–227.3); in men, 195.7 per 100,000 person-years (95% *CI*: 142.4–249.0) and in women, 188.1 per 100,000 person-years (95% *CI*: 138.6–237.7).

The latest research indicates that traditional risk factors for stroke peak in adults aged 35–44, accounting for nearly 33% of strokes in men and approximately 40% in women (Swartz, 2024).

According to the Global Burden of Disease 2016 Lifetime Risk of Stroke Collaborators report, the global average lifetime risk of stroke increased from 22.8% in 1990 to 24.9% in 2016, a relative increase of 8.9% (95% *CI*: 6.2%−11.5 %). This increase is attributed to an aging population and increasing risk factors, which contribute to a higher lifetime risk of stroke (Virani et al., 2020).

The Get with the Guidelines (GWTG) stroke registry includes cases from 41 hospitals in Colorado since 2010. The most common cardiovascular risk factors were hypertension, hyperlipidemia, and smoking in men, and hypertension, smoking, and obesity in women. The most common non-cardiovascular risk factors included migraines, renal impairment, and thrombophilia in men, and migraines, malignancy, and thrombophilia in women (Leppert et al., 2023).

Historically, stroke has been primarily associated with older adults; however, clinical and scientific interest in ischemic stroke in young adults has increased significantly in the last decade. In Honduras, data on stroke prevalence in different regions for older adults have been reported, but information on incidence and characteristics in young adult patients is limited. This lack of data hinders a full understanding of the impact and specificities of this population, limiting the ability to provide adequate care for this group. The objective of this study was to determine the characteristics and factors associated with stroke in young adult patients treated at a tertiary hospital in Honduras.

## Methods

### Study design

An observational, hospital-based cross-sectional study was conducted among patients aged 18–55 years who presented to the emergency department of a tertiary public hospital in Tegucigalpa, Honduras, with a diagnosis of stroke between January and July 2024. A non-probability convenience sampling strategy was used, and 70 eligible patients were recruited during the study period. Given the cross-sectional design and sampling approach, the study was intended to describe clinical characteristics, diagnostic findings, stroke sub-types, and frequent risk factors in this population, rather than to establish causal relationships or independent predictors of stroke.

### Participants and sampling

Eligible participants were patients aged 18–55 years, of either sex, with a clinical diagnosis of stroke confirmed by neurological evaluation and neuroimaging when available. Patients were recruited using a non-probability convenience sampling strategy. This approach was adopted because recruitment depended on the availability of eligible patients during the study period and the feasibility of completing clinical and diagnostic assessments in a resource-limited tertiary-care hospital setting. A total of 70 eligible patients were included.

### Age definition

Young adult stroke was defined as stroke occurring in patients aged 18–55 years. This cutoff was selected because there is no universally accepted age threshold for young-onset stroke, and previous studies have used upper age limits ranging from approximately 45 to 59 years. The incidence of ischemic stroke has increased alarmingly among young adults (18–49 years old) in recent years in high-income countries, in contrast to the decline in older populations (Rasing et al., 2026).

## Data collection and variables

Information was obtained through interviews with patients or family members and from clinical records. The questionnaire included sociodemographic characteristics and clinical history, including hypertension, diabetes mellitus, chronic kidney disease, smoking, alcohol consumption, heart disease, migraine, cancer, epilepsy, previous cerebrovascular events, miscarriages, sickle cell anemia, physical inactivity, obesity, oral contraceptive use, and illicit drug use.

## Clinical and diagnostic assessment

Patients presenting with stroke symptoms underwent neurological evaluation. Stroke severity was assessed using the National Institutes of Health Stroke Scale. Functional status was assessed using the modified Rankin Scale (mRS) at hospital discharge. Non-contrast brain computed tomography was performed in most patients, and magnetic resonance imaging or CT angiography was performed when clinically available. Ischemic and hemorrhagic stroke subtypes were classified according to clinical and neuroimaging findings. For ischemic stroke, presumed etiology was categorized according to available clinical, vascular, cardiac, laboratory, and imaging information. Cases without sufficient etiologic evaluation were classified as indeterminate or incompletely evaluated.

Short-term clinical outcomes were assessed during the index hospitalization. Mortality was defined as in-hospital death occurring before discharge. Functional status was assessed at hospital discharge using the modified Rankin Scale, according to the information available in the clinical record. No post-discharge follow-up assessment was performed.

The project was approved by the Biomedical Research Ethics Committee (CEIB) at Faculty of Medical Science of National Autonomous University of Honduras, approval code: 079-2024. The privacy and confidentiality of the participants were guaranteed. Informed consent was obtained before data collection and clinical procedures.

## Measures

Patients who presented to the emergency room with stroke symptoms were evaluated neurologically, the National Institute of Health Stroke Scale (NIHSS) scale was applied to determine the severity of the stroke, computed axial tomography and the radiological evaluation of the extension of stroke Alberta Stroke Program Early CT Scores (ASPECTS) scale, in a subgroup of patients CT angiography and brain magnetic resonance imaging were performed. For transient ischemic attacks, the ABCD2 scale was used to determine the risk of stroke. The Modified Rankin Scale was used for functional assessment after a cerebrovascular event (Powers et al., 2019). For hemorrhagic strokes, the ICH scale (*Intracerebral Hemorrhage Score*) was used to prognostically determine the mortality rate in patients (Hemphill et al., 2001).

The information was complemented with clinical examinations that included laboratory tests (CBC, Glycosylated hemoglobin, lipid profile, HIV), carotid Doppler and transcranial Doppler with bubble test for the detection of right-left shunt in patients with stroke in young patients who do not have an identifiable cause of the stroke, at the time of the corresponding evaluation.

## Statistical analysis

Data was analyzed using SPSS version 26 (license acquired by the IP). Categorical variables were summarized as frequencies and percentages, whereas continuous variables were summarized using means and standard deviations or medians and interquartile ranges, according to their distribution. No formal sample size calculation was performed before recruitment, hospital-based cross-sectional design, the non-probability convenience sampling strategy, the limited sample size, and the absence of a non-stroke control group meant that the analysis was primarily descriptive. Percentages were calculated according to the relevant denominator, which is specified for each subgroup analysis.

## Results

Of the 70 patients studied, 61.4% of patients were women; the mean age was 43.4 ± 9.1 years. The median age was 46 years (IQR: 40–49 years); 97.1% were mestizo; and 37.1% had completed primary education. The most reported occupation was housekeeper.

Of all patients evaluated, 55.7% (*n* = 39) had a history of high blood pressure, 31.4% (*n* = 22) had type 2 diabetes mellitus, and 24.3% (*n* = 17) had a history of migraine. Of the women evaluated, 12.9% (*n* = 9) had a history of previous abortions.

Regarding the modifiable risk factors present, 42.9% (*n* = 30) were physically inactive, 22.9% (*n* = 16) were obese class II, 21.4% (*n* = 15) were smokers, and 18.6% (*n* = 13) were frequent alcoholics. The 12.9% (*n* = 9) of the patients had obstructive sleep apnea, and 15.7% (*n* = 11) of the women reported using oral contraceptives (Table 1).

**Table 1:** Clinical history, comorbidities, and modifiable risk factors among young adult patients with stroke, Hospital Escuela, in the period January–July 2024, *n* = 70.

Background	*n*	%
In-hospital days (mean ±*SD*)	5.5 ± 2.6	
Comorbidities		
Chronic arterial hypertension	39	55.7
Time of evolution of hypertension (mean ±*SD*)	5.0 ± 5.2	
Diabetes mellitus	22	31.4
Time of evolution of DM (mean ±*SD*)	5.4 ± 4.1	
Chronic kidney disease	4	5.7
Time of evolution of CKD (mean ±*SD*)	4.8 ± 3.7	
Heart disease	7	10
Time of evolution of heart disease (mean ±*SD*)	9.5 ±14.3	
Migraine	17	24.3
Cancer	5	7.1
Epilepsy	5	7.1
Previous stroke	4	5.7
Time course of previous stroke (mean ±*SD*)	6.1 ± 9.3	
Abortions	9	12.9
Sickle cell anemia	1	1.4
Acute lymphocytic leukemia	1	1.4
Associated factors		
Physical inactivity	30	42.9
Dyslipidemia	5	7.1
Body mass index		
Overweight (BMI 25–29.9 kg/m^2^)	12	17.1
Obesity class I (BMI 30–34.9 kg/m^2^)	10	14.3
Obesity class II (BMI 35–39.9 kg/m^2^)	16	22.9
Obesity class III (BMI 40–49.9 kg/m^2^)	5	7.1
Abdominal obesity^+^	35	50
Smoking	15	21.4
Alcoholism	13	18.6
Drug use	8	11.4
Obstructive sleep apnea	9	12.9
Use of oral contraceptives	11	15.7

SD, standard deviation; ^+^abdominal obesity was defined as waist circumferences ≥102 cm for men and ≥88 cm for women.

The most frequent onset of symptoms was within 9–24 h, in 38.6% (*n* = 27) of cases. Symptoms appeared suddenly in 82.9% (*n* = 58) of cases. The most common clinical manifestation was hemiparesis or hemiplegia in 84.3% (*n* = 59) of patients, followed by dysarthria in 44.6% (*n* = 31) and altered consciousness in 38.6% (*n* = 27). The most frequent clinical syndrome was middle cerebral artery syndrome in 51.4% (*n* = 36) of cases. Most patients had a NIHSS score in the range of 5–15 points (moderate) in 42.9% (*n* = 30), and a mean score of 9.4 ± 6.7 (Table 2).

**Table 2:** Clinical presentation, symptom to admission interval, and neurological severity among young adult patients with stroke, Hospital Escuela, January–July 2024, *n* = 70.

Clinical manifestations	*n*	%
Evolution time		
0–60 min	1	1.4
1–4.5 h	8	11.4
4.5–6 h	10	14.3
6–9 h	5	7.1
9–24 h	27	38.6
24–48 h	11	15.7
Greater than 48 h	8	11.4
Appearance of symptoms		
Sudden	58	82.9
Progressive	12	17.1
Symptoms and signs		
Hemiparesis or hemiplegia	59	84.3
Dysarthria	31	44.3
Alteration of consciousness	27	38.6
Hemihypoesthesia or hemianesthesia	19	27.1
Alteration of conjugate gaze	16	22.9
Aphasia	14	20
Altered visual fields	12	17.1
Vertigo or ataxia of the extremities	9	12.9
Clinical syndrome		
Ischemic stroke		
Middle cerebral artery syndrome	36	51.4
Lacunar syndromes	7	10
Cerebellar syndrome	6	8.6
Transient ischemia attack (TIA)	1	1.4
*Hemorrhagic CVA*	20	28.6
NIHSS		
0 without stroke	2	2.9
1–4 (minor stroke)	7	10
5–15 (moderate stroke)	30	42.9
16–20 (moderate to severe stroke)	7	10
21–42 (severe stroke)	5	7.1
NIHSS score (*x* ±*SD*)	9.4 ± 6.7	
ABCD/2		
0–3 low risk	1	1.4

NIHSS, National Institutes of Health Stroke Scale.

Non-contrast brain CT was performed in 98.6% (*n* = 69/70) of patients, whereas brain MRI in 11.4% (*n* = 8), and CT angiography in 5.7% (*n* = 4). Ischemic stroke was identified in 71.4% (*n* = 50) cases, of which 61.4% (*n* = 43) were non-lacunar strokes and 10% (*n* = 7) were lacunar strokes. The ASPECT score was calculated with a mean of 7 ± 2. In addition, hemorrhagic stroke was detected in 28.6% (*n* = 20) of patients, with a mean ICH score of 3 (72% probability of 30-day mortality).

Leukocytosis was found in 17.1% (*n* = 12) of patients, elevated nitrogen levels in 8.6% (*n* = 6) and hyperglycemia in 5.7% (*N* = 4). These patients underwent glycosylated hemoglobin testing, finding elevated values in 8.6% (*n* = 6), with a mean of 9.6 ± 2. Abnormalities in the lipid profile were detected in 8.6% (*n* = 6) of patients. In 41.4% of patients (*n* = 29) these laboratory parameters were not performed.

Electrocardiogram abnormalities were identified in 17.1% (*n* = 12) of patients: atrial fibrillation 11.4% (*n* = 8), acute myocardial infarction 2.9% (*n* = 2), left ventricular hypertrophy and sinus bradycardia 1.4% (*n* = 1), in 5.7% EKG was not performed. The echocardiogram was performed in 12.9% (*n* = 9) of patients, finding ischemic heart disease in 5.7% (*n* = 4), hypertensive heart disease in 2.9% (*n* = 2), and left ventricular hypertrophy, valvular insufficiency, and dilated heart disease in 1.4% (*n* = 1). Carotid Doppler ultrasound was performed in 25.8% (*n* = 18) of patients, with abnormalities in 2.9% (*n* = 2); 24-h Holter monitoring was not performed.

In a subgroup of patients (*n* = 17) with cryptogenic stroke, transcranial Doppler with bubble test was performed, of which 58.8% (*n* = 10) were female, mostly with middle cerebral artery stroke and cerebellar stroke and who presented a Risk of Paradoxical Embolism (RoPE) of 7 ± 1, a positive result for right-to-left shunt due to patent foramen ovale (PFO) was obtained in 29.4% (*n* = 5) of the patients, 7.1% of the total sample (see Table 3).

**Table 3:** Diagnostic findings among cryptogenic stroke patients who underwent transcranial Doppler with bubble testing, Hospital Escuela, January–July 2024, *n* = 17.

Patient characteristics	*n*	%
**Age (*****x*** **±*****SD*****)**	42 ± 11	
Sex		
Female	10	58.8
Male	7	41.2
Type of stroke		
Middle cerebral artery stroke	10	58.8
Cerebellar stroke	5	28.4
Posterior cerebral artery stroke	1	5.9
TIA	1	5.9
Electrocardiogram		
Normal	17	100
Echocardiogram		
Normal	9	52.9
Not performed	8	47.1
Carotid Doppler USG		
Normal	12	70.6
Not performed	5	29.4
Glycosylated hemoglobin and lipid profile		
Abnormal	3	17.6
Normal	14	82.4
Transcranial Doppler results		
Risk of paradoxical embolism (RoPE) (*x* ±*SD*)	7 ± 1	
Right-left shunts		
Negative	12	70.6
Positive	5	29.4

## Identification of the type of stroke in a young patient and its etiology

A total of 71.4% (*n* = 50) of patients were identified as having ischemic stroke; of these, 61.4% (*n* = 43) were non-lacunar strokes. The primary etiology was cardioembolic in 21.4% (*n* = 15), followed by small vessel disease in 7.1% (*n* = 5), and 34.3% (*n* = 24) had an incomplete or undetermined evaluation.

Hemorrhagic stroke was found in 28.6% (*n* = 20) cases, with arterial hypertension being the main etiology in 22.9% (*n* = 16) and arteriovenous malformations and amyloid angiopathy in 2.9% (*n* = 2) (see Table 4).

**Table 4:** Stroke subtype and presumed etiologic classification among young adult patients with stroke, Hospital Escuela, January–July 2024, *n* = 70.

Characteristics	*n*	%
Types of stroke		
Hemorrhagic	20	28.6
Ischemic	50	71.4
Type of ischemic stroke		
Lacunar	7	10
Not lacunar	43	61.4
Etiology		
Cardioembolic	15	21.4
Small vessel disease	5	7.1
Determined etiology	4	5.7
Atherosclerosis of long arteries	2	2.9
Indeterminate/incomplete evaluation	24	34.3
Type of hemorrhagic stroke		
Intracerebral hemorrhage	20	28.6
Etiology of intracerebral hemorrhage		
High blood pressure	16	22.9
Amyloid angiopathy	2	2.9
Arteriovenous malformation	2	2.9

Indicated treatment included ASA in 64.3% (*n* = 45) of patients, atorvastatin in 70.0% (*n* = 49), clopidogrel in 4.3% (*n* = 3), therapeutic anticoagulation in 5.7% (*n* = 4), labetalol in 24.3% (*n* = 17), and mannitol in 20.0% (*n* = 14). During the index hospitalization, hospital mortality was 15.7% (*n* = 11). Among patients discharged alive, functional status at discharge showed that 21.4% (*n* = 15) had severe disability, 27.1% (*n* = 19) had moderate disability, 7.1% (*n* = 5) had mild disability, and 11.4% (*n* = 8) had very mild disability.

## Discussion

The objective of this study was to describe the clinical characteristics, diagnostic findings, stroke subtypes, and frequent risk factors among young adult patients with stroke treated at a tertiary care hospital in Honduras. This study is of great importance, given that cerebrovascular disease represents an incidence of between 3.6 and 5.7 per 1,000 inhabitants in rural and urban communities, respectively, in the country (Hernández Mejía et al., 2021).

Regarding sociodemographic characteristics, a higher number of stroke cases were found in women, with a mean age of 43.4 years, a predominance of mixed-race patients, and a primary education level. The most reported occupation was housekeeper. These findings contrast with a retrospective descriptive study conducted at the Escuela Hospital, which reported a mean age of 30 years (lower than that found in this study), with 72.8% of the cases being women. In that study, hemorrhagic stroke was more frequent (51.4%), with the intracerebral subtype predominating (28.6%). The most significant comorbidities were high blood pressure (20.0%), obesity (12.8%), and alcoholism (8.6%) (Mejía et al., 2016).

The study identified high blood pressure, type 2 diabetes mellitus, and migraine as predominant comorbidities. Furthermore, 21.4% of the patients were smokers, and 15.7% of the women used oral contraceptives. Hypertension was highlighted as a common factor in both studies conducted in Honduras.

Leppert et al. (2022), in a systematic review of sex differences in ischemic stroke in young adults, found that women younger than 35 years of age had 44% more cases of ischemic stroke compared to men. This difference narrows in the 35–45 age group, where the gender gap is narrowest. Furthermore, there is conflicting evidence as to whether men or women predominate in this age group with ischemic stroke.

As reported by Ekker et al. (2019) the incidence of any cerebrovascular event in young people increases with age in those over 35 years of age, is higher in women than in men aged 18–44 years and has increased by 23% in a decade, at the expense of ischemic stroke. This finding contrasts with that reported by Ma et al. (2024), who observed an increase in stroke incidence in the 30- to 39-year-old age group, demonstrating an emerging trend toward younger ages. Based on these trends, it is necessary to intensify preventive efforts, promote healthy lifestyles, strengthen the health system, and maintain rigorous epidemiological monitoring.

In a cross-sectional study conducted at a comprehensive center in Bogotá, Colombia, patients younger than 50 years of age who suffered an ischemic stroke between 2011 and 2018 were evaluated. A descriptive analysis of comorbidities, the Trial of Org for Acute Stroke (TOAST) classification, the National Institute of Health Stroke Scale (NIHSS), and the modified Rankin Scale (mRS) were performed. A total of 152 patients were included, of whom 50.66% were men. The most frequent risk factors were a history of smoking (19%), high blood pressure (18%), cardiovascular disease (17%), and migraine (15%) (Aguilera-Pena et al., 2021).

The incidence of stroke is higher in patients with type 2 diabetes and in non-diabetics with insulin resistance, which is associated with increased morbidity and mortality. The main risk factors for stroke in patients with type 2 diabetes include age, hypertension, metabolic syndrome, diabetic nephropathy (in both type 1 and type 2 diabetes), peripheral and coronary artery disease, and the presence of atrial fibrillation (Bell and Goncalves, 2020).

This study found a high proportion of patients with a history of migraine (24.3%). This finding is consistent with that reported by Aguilera, who identified that 15% of participants had a history of migraine (Aguilera-Pena et al., 2021). This result coincides with a meta-analysis conducted by Schürks et al. (2009), which revealed that patients with migraine have a 1.73-fold higher risk (95% CI: 1.31–2.29) of developing ischemic cerebrovascular disease, with an increased risk of up to 2.08-fold in women. This is attributed to the fact that migraine predisposes patients to endothelial dysfunction and increased platelet activation and aggregation. Neuroimaging studies have revealed a higher prevalence of asymptomatic structural brain lesions in people with migraine (Øie et al., 2020).

Smoking was present in 21.4% of patients. A meta-analysis by Pan et al. (2019), which included 14 studies with 303,134 subjects, found that smokers had a higher overall risk of stroke compared with non-smokers, with a pooled odds ratio (OR) of 1.61 (95% *CI*: 1.34–1.93; *p* < 0.001). The association between stroke of any type and smoking was also statistically significant. Smokers had a higher risk of stroke compared with non-smokers (*OR*: 1.46; 95% *CI*: 1.04–2.07; *p* < 0.001), with an influence of sex: in men, OR: 1.54 (95% *CI*: 1.11–2.13; *p* = 0.002), and in women, OR: 1.88 (95% *CI*: 1.45–2.44; *p* < 0.023).

Regarding illicit drug use, the study found that 11.4% of patients reported using it. Bukhari et al. (2023) noted that illicit drug use is the fifth most common factor associated with ischemic stroke, with cerebral vasospasm being the main pathogenetic mechanism in cocaine users. The risk of stroke can be reduced through lifestyle changes, such as regular exercise and a diet rich in fruits and vegetables (Higgins, 2024).

In this study, it was observed that the onset of symptoms before arrival at the hospital presented an evolution between 9 and 24 h in 38.6% of cases, which contrasts significantly with the study by Tancredi et al. (2013), in which 33% of patients experienced a temporary delay of less than 3 h and only 21% presented an evolution between 6 and 24 h (Tancredi et al., 2013).

The predominant clinical manifestations in the study, such as hemiparesis or hemiplegia, together with the high frequency of sudden onset of symptoms (82.9%), are consistent with the classic characteristics of ischemic stroke in the general population. However, this pattern contrasts with other atypical presentations also observed in this age group, such as those described by Singhal et al. (2013).

Regarding the NIHSS classification upon admission, 42.9% of patients presented with a moderately severe stroke. This finding contrasts with that reported by Matuja et al. (2020), who found that 43.9% of patients presented with a severely severe stroke upon admission. Neuroimaging results showed that most cases corresponded to ischemic stroke (71.4%).

The identification of cardioembolic stroke as the main etiology in 21.4% of the cases in this study is comparable with that reported by Namaganda et al. (2022), who, in a study conducted in 51 patients under 59 years of age, found that 76.5% had ischemic stroke. This contrasts with that described by Aguilera-Pena et al. (2021), who reported the most frequent etiology to be the cardioembolic event, representing 23.6% of cases.

The incidence of ischemic stroke in young adults is increasing, representing a public health problem. The causes of ischemic stroke in this group can be classified into five categories: cardioembolism, large-vessel vascular disease, small-vessel vascular disease, toxic-metabolic disorders, and hypercoagulability (Mei and Schaefer, 2023).

Cardioembolic stroke was the most frequently presumed defined etiology in this cohort, this finding should be interpreted with caution because a substantial proportion of ischemic stroke cases had an indeterminate or incomplete etiologic evaluation. Incompletely classified cases likely reflect limited access to comprehensive etiologic testing. Therefore, the true etiologic distribution of young onset stroke in this setting remains uncertain and should be further investigated in future studies.

Within the study, a subgroup of 17 patients with cryptogenic stroke underwent transcranial Doppler with bubble testing, identifying a patent foramen ovale in 5 patients (29.4%) of the tested subgroup and 7.1% of the total sample. This finding contrasts with that described by Potter et al. (2022), who in a recent review suggested that this condition is present in up to 27% of the general population, with variably reported sex differences (Potter et al., 2022). Among cryptogenic strokes in young adults, the presence of a patent foramen ovale (PFO) has been found in 40–56% of cases (Alsheikh-Ali et al., 2009; Handke et al., 2007). The findings reported in the study show a lower, but significant, prevalence associated with the etiology of stroke in young patients.

Paradoxical embolism through a patent foramen ovale (PFO) is a common cause of ischemic stroke, accounting for approximately 1 in 20 cases of all ischemic strokes (Saver, 2024).

The mortality observed in the study was 15.7%, lower than that reported by Matuja et al. (2020), who recorded a mortality of 49.1% in young adults. There is a high burden of stroke in this age group, associated with a high 30-day mortality rate. Patients were left with some degree of disability, affecting the economically active population, which emphasizes the severity of stroke in this group and the need to implement more effective therapeutic strategies. It should be noted that patients who were candidates for thrombolysis could not be offered this therapy due to the lack of availability of the drug in the hospital.

In 2019, the age-standardized stroke-related mortality rate was 3.6 (95% *CI*: 3.5–3.8) times higher in low-income countries compared to high-income countries, according to the World Bank. Similarly, the age-standardized stroke-related disability-adjusted life-year (DALY) rate was 3.7 (95% *CI*: 3.5–3.9) times higher in low-income countries compared to high-income countries (Feigin et al., 2021). A strength of this study is the comprehensive clinical characterization of young adult patients with stroke, including neurological severity, imaging-based stroke classification, vascular and cardiac evaluation, targeted PFO screening, and short-term functional outcomes.

The high frequency of modifiable vascular and lifestyle related factors observed in this hospital-based cohort suggests several potential areas for local prevention. Hypertension screening, Diabetes prevention and control strategies. The high frequency of physical inactivity and obesity also supports the need for community-based interventions among women, special attention should be given to the combined presence of migraine, smoking, hypertension, obesity, and oral contraceptive use.

These findings do not establish causal relationships, but they highlight clinically relevant factors observed among young adult patients with stroke and may help guide local prevention strategies in Honduras.

## Limitation

This study has several limitations. First, the single-center hospital-based design and non-probability convenience sampling may have introduced selection bias and limit the generalizability of the findings to all young adults with stroke in Honduras. Second, because of the cross-sectional design, temporality and causality cannot be established. Therefore, the clinical and lifestyle-related factors reported in this study should be interpreted as frequent characteristics observed in the enrolled patients, rather than as independent causal predictors of stroke. Third, the absence of a control group and the limited sample size restricted the ability to perform robust multivariable analyses.

The study did not include a non-stroke comparison group; therefore, odds ratios for stroke occurrence and independent predictors of stroke could not be estimated. The descriptive nature of statistical analysis limits causal inference and prevents confirmation of independent associations between the evaluated clinical factors and stroke. Future multicenter studies with larger samples and appropriate control groups should incorporate multivariable regression models with ORs, 95% confidence intervals, and p-values to better assess independent risk factors for young-onset stroke in Honduras.

Another important limitation was the incomplete coverage of etiologic diagnostic tests. Echocardiography, carotid Doppler ultrasound, prolonged cardiac rhythm monitoring, and transcranial Doppler with bubble testing were performed only in selected patients rather than systematically across the entire sample. This may have limited the accuracy of etiologic classification and contributed to the proportion of cases categorized as indeterminate or incompletely evaluated. Therefore, the etiologic findings should be interpreted with caution. Future studies should apply standardized diagnostic protocols to improve classification of stroke mechanisms in young adults.

Because no post-discharge follow-up was performed, this study cannot assess 30-day mortality, 90-day functional outcomes, stroke recurrence, rehabilitation progress, or long-term disability.

All data was collected from a single hospital using convenience sampling, a total of 70 patients were recruited over a 7-month period, this limited sample size reduced statistical power and restricted the possibility of performing robust inferential analyses or multivariable regression models. The cohort may not represent all young adults with stroke in Honduras. Patients with mild or transient symptoms who did not seek hospital care, patients managed in other healthcare facilities, and patients who died before reaching the hospital were not captured. As a result, the observed clinical severity, mortality, and disability rates may reflect the profile of patients who reached a tertiary referral hospital rather than the full spectrum of young-onset stroke in the community. Therefore, the study's findings cannot be extended to young adult stroke patients across Honduras or the wider regional population.

In conclusion, stroke in young adults in the Honduran population is a public health problem with a high incidence rate in women, and a predominance of ischemic strokes of cardioembolic etiology. Modifiable risk factors, such as high blood pressure, diabetes mellitus, abdominal obesity, and physical inactivity, were prevalent in this group, suggesting a significant window for preventive interventions. These findings support the need for locally adapted prevention strategies aimed at early detection and control of vascular risk factors in young adults. In patients with cryptogenic stroke, these factors were associated with the presence of patent foramen ovale.

Although these findings are derived from a single tertiary hospital and should not be interpreted as population-level estimates, they provide relevant local evidence on young-onset stroke in Honduras and may support the development of future multicenter studies and targeted public health interventions.

## Data Availability

The original contributions presented in the study are included in the article/supplementary material, further inquiries can be directed to the corresponding author.
